# The effect of destination personality dimensions on park visitors’ pro-environmental behavior: an application of stimulus-organism-response model

**DOI:** 10.3389/fpsyg.2026.1734312

**Published:** 2026-04-02

**Authors:** Guofang Shi, Haili Shen, Huwen Liu

**Affiliations:** 1 Liangshan College, Lishui University, Lishui, Zhejiang, China; 2 Department of Tourism and Hospitality Management, School of Management, Zhejiang University, Hangzhou, Zhejiang, China; 3 International School of Cultural Tourism, Hangzhou City University, Hangzhou, China

**Keywords:** forest recreation, park personality, park visitation, place attachment, PLS-SEM, pro-environmental psychological mechanism, self-congruity

## Abstract

**Introduction:**

The sustainable development of forest parks is closely associated with the conservation and preservation of natural resources and landscape environments. Stimulating visitors to be involved in spontaneous on-site Pro-Environmental Behavior (PEB) has been recognized as an important strategy to enhance ecological sustainability. Drawing on the stimulus-organism-response (SOR) model and destination personality theory, this study develops a conceptual model illustrating the mechanism underlying the relationship between destination personality of park (park personality) and PEB.

**Methods:**

A total of 785 effective responses were gathered at an iconic forest park-Banshan national forest park (BNFP) in Hangzhou, China. Using both qualitative and quantitative research methods, five underlying dimensions of forest park’ destination personality were uncovered, namely genuine, competent, sophisticated, exciting and tranquil.

**Results:**

PLS-SEM analysis revealed that destination personality dimensions of forest park vary in their effects on visitors’ self-congruity and place attachment. The findings also provide empirical evidence for the influence of self-congruity and place attachment on visitors’ on-site PEB. Furthermore, self-congruity significantly mediates the link between three park personality dimensions (genuine, exciting and tranquil) and PEB. The findings also highlight the role of place attachment as a mediating variable between three dimensions of park personality (competent, exciting and tranquil) and visitors’ PEB intention.

**Discussion:**

This study provides practical implications for fostering the sustainable development of forest parks.

## 1 Introduction

The tourism industry, which has consistently outpaced global economic growth, now faces the urgent task of minimizing its environmental footprint (Li and Wu, 2020; Qiu et al., 2023). Sustainable tourism is inseparable from the protection and preservation of natural environments, especially in nature-based destinations whose attractiveness and competitiveness hinge on the quality of their environmental assets (Wu et al., 2021). This imperative has intensified in the post-COVID era, as the pandemic triggered a surge in demand for outdoor and nature-based experiences that allow travelers to maintain physical distance and feel safe (Johnson et al., 2023). While such tourism has injected vital income into local economies, it has also amplified environmental pressures—over-crowding, pollution, and habitat destruction foremost among them (Wang, 2019). These problems are now so widespread and severe that they threaten the ecological integrity of destinations across the globe (Gundersen et al., 2024). A primary driver of this degradation is the irresponsible on-site behavior of visitors, whose cumulative actions are steadily eroding the very natural beauty and distinctiveness that drew them there (Aziz and Niazi, 2023; Li and Wu, 2020). Unless these impacts are decisively addressed, environmental decline will undermine the long-term sustainability of tourism in nature-based destinations, making the need for mitigation more pressing than ever.

Destination personality has emerged as a prominent and crucial theory in the design and positioning of destination offerings within natural areas (Zhang and Bai, 2011). This concept is intrinsically related with the affective element of destination image. Prior research has corroborated that destination personality can capture the softer, inherent attributes of a location and reflect symbolic meanings deeply embedded in tourist behavior (Ekinci and Hosany, 2006; Hosany et al., 2006). For tourism places, establishing a set of distinct personality traits is crucial to attracting visitors and generating tourism revenues (Chi et al., 2018). As a formidable marketing tool, destination personality enables destination operators to differentiate themselves from competitors and gain a competitive advantage in the leisure and tourism industry. Destination personality is hypothesized to rapidly foster the emotional connections between tourists and travel destinations (Zhang et al., 2019). A distinctive personality implies unique and enduring traits that encourage emotional bonding between consumers and tourism products (Tran et al., 2013), which in turn leads to more positive product evaluations (Li et al., 2014). Further studies suggest that destination personality constitutes a pivotal factor in predicting visitors’ future behavioral intentions (Papadimitriou et al., 2015).

Forest parks deliver substantial environmental benefits, contributing critically to the environment, renewable energy, ecosystem services and human wellbeing (Li et al., 2021). They serve multiple roles in contemporary society. As “social equalizers,” they help reduce mortality rates when visitors form an emotional bond with the natural environment (Krapez et al., 2021). Forest parks also act as venues to mitigate mental fatigue, provide opportunity for self-discovery, and nurture perception psychological wellbeing (Seely, 2022). From the vantage of destination personality, forest park professionals can enhance consumption value, create pleasant memoirs, and reinforce visitor satisfaction (Shi et al., 2025). A distinctive and coherent destination personality is hypothesized to foster emotional connections between park visitors and the destinations (Kim and Stepchenkova, 2017), thereby promoting more positive on-site behaviors (Sharifsamet et al., 2020). Arguably, strengthening destination personality is crucial for forest parks to achieve long-term environmental sustainability. However, prior studies on visitor behavior in parks, natural environments and other eco-tourism destinations have focused primarily on visitors’ evaluation of the functional clues and substantive attributes of tourism offerings (Kim et al., 2021). Such research offers limited insight into the symbolic meanings that visitors attach to their visitation experiences, as well as the role of these intangible attributes in shaping visitors’ attitudes and behaviors.

An important strategy for advancing sustainable tourism is to encourage visitors to engage in on-site Pro-Environmental Behavior (PEB) (Lin et al., 2022). Nurturing PEB among visitors has been recognized as a vital sustainable tourism practice for achieving symbiotic relationships between visitors and destination environment (Li et al., 2025). Existing research on the antecedents of PEB has relied primarily on individual-level factors, such as demographic characteristics and socio-psychological factors, including environmental awareness (Han and Hyun, 2017; Wang et al., 2021) and face consciousness (Wu et al., 2022) Thus, there is a lack of empirical studies about visitors’ perceptions of forest parks’ destination personality, let alone how such perceptions shape visitors’ PEB intentions.

The destination environment serves as a reservoir for the emotional bonds that visitors form (Davis, 2016). Destination personality has been conceptualized as a critical element of place-based experience. Quest for unique spiritual experience is one of the essential motivational forces for visitors to forest destinations (Bosworth and Curry, 2020). Self-congruity is an important component of self-concept, referring to the extent to which an individual’ self-concept aligns with the perceptions and feelings aroused by objects, events, and, of course, places (Boley et al., 2022). Place attachment, on the other hand, represents an affective attitude or sentiment that people hold toward the environment (Dwyer et al., 2019). These two concepts constitute the internal organismic processes for park visitors, thus functioning as two central mediating pathways in the relationship between park personality and visitors’ PEB. However, the research team is not aware of any studies examining how different dimensions of destination personality influence visitors’ PEB intention in the context of forest parks. Nor is it aware of any work elucidating the psychological mechanism underlying park visitors’ PEB.

This study aims to address the aforementioned research gap by integrating the concept of destination personality to explore the symbolic attributes of forest park. Specifically, this study employs the stimulus-organism-response (SOR) model to investigate the formation mechanism underlying visitors’ PEB in the context of forest parks. The specific research approach is as follows: (1) To test the main effect model encompassing the direct relationships among destination personality, place attachment, self-congruity, and PEB; (2) To elucidate the mediating roles of self-congruity and place attachment in the relationship between destination personality and PEB. Theoretically, this study enriches the literature by examining visitors’ PEB from the perspective of destination personality. Practically, it provides implications for cultivating vigorous emotional connections between visitors and forest parks, promoting visitors’ PEB, and enhancing the ecological sustainability of forest parks. Thus, this study provides a valuable reference for the development and marketing of forest park destinations.

## 2 Theoretical framework

### 2.1 Stimulus-organism-response model

The stimulus-organism-response (SOR) model is a paradigm that crystallizes the intrinsic associations among three fundamental elements: environmental antecedents, internal states, and behavioral response (Mehrabian and Russell, 1974). The SOR model proposes that the external factors (S) within the ambient environment trigger internal cognitive and affective processes (O), which further generate subsequent attitudes and behaviors (R). This sequence forms a psychological process that underpins individuals’ decision-making (Wu and Li, 2018). The SOR model has been widely applied and validated in research on tourist behavior and leisure involvement (e.g., Zhang and Xu, 2019; Su and Swanson, 2019). This framework has been identified as an insightful tool for probing into the issue of sustainable tourism (Chakraborty et al., 2024; Wu and Wang, 2025).

This study extends the application of the SOR framework to the context of forest parks, focusing on the interaction between actual visitors and the softer, inherent traits of park destinations (i.e., park personality). This theoretical framework provides a multi-layered perspective for comprehending the mechanism by which visitors’ perceptions of park personality (external stimulus) are translated through cognitive characteristics (self-congruity) and affective state (place attachment) into behavioral intention (PEB).

### 2.2 Destination personality

Personality systematically reflects individuals’ relatively stable styles of cognition, affect and behavior (McCrae and Costa, 1997). In the marketing literature, brand personality refers to the tendency for consumers to attribute human personality traits to a brand (Aaker, 1996; Sweeney and Brandon, 2006). This concept of brand personality was applied in destination branding. This concept has been extended to destination branding research, giving rise to the notion of destination personality, which captures the human-like traits attributed to a destination (Hosany et al., 2006). Destination personality is formally defined as “the set of human characteristics associated with a destination” (Ekinci and Hosany, 2006).

According to the theories of anthropomorphism and symbolism, destination personality is regarded as a viable metaphor that personifies the intangible attributes and intrinsic characteristics of a destination (Aaker and Fournier, 1995). It represents a collection of all non-functional, symbolic, and experiential characteristics of a place (Hankinson, 2005). Concerning the relationship between destination personality and destination image, scholars have argued that destination personality should be viewed as a sub-concept of destination image. Destination image has been recognized to comprise both cognitive and affective components (San Martín and del Bosque, 2008). These researchers regarded destination personality as the affective side of destination image (Murphy et al., 2007b; Xie and Lee, 2013).

Further studies suggest that destination personality represents a pivotal factor in examining tourists’ attitudes and evaluation toward a destination. Several empirical findings indicate that destination personality operates as a self-expressive function that strengthens tourists’ destination preferences (Kovačić et al., 2022; Zacharia and Spais, 2017), identification (Hultman et al., 2015), and sense of ownership toward specific destination (Kumar and Nayak, 2019). Furthermore, existing literature highlights that destination personality acts as a catalyst in strengthening visitors’ pleasurable memories of their destination experience, which in turn consolidates their revisit intention (Kim et al., 2018; Yang et al., 2020) and fosters favorable attitudes and behavioral responses toward the destination (Huang et al., 2017; Wu and Lai, 2023).

Park personality refers to the diverse destination personalities associated with a single park destination, rooted in brand personality theory and supported by the theories of anthropomorphism and symbolism (Zhang et al., 2019). Developing comprehension about these human-like traits is important for parks to achieve environmental sustainability. The concept of park personality can act as a proxy variable to elucidate the heterogeneity embedded within park visitors’ behavior and discern how visitors make park choices (Quintal et al., 2019; Quintal et al., 2024). Currently, there is a lack of empirical research on park personality. In the context of botanic parks, two principal dimensions of personality traits were identified: excitement and competency (Quintal et al., 2019). Krapez et al. (2021) identified another two crucial dimensions–natural and manicured–that define the personality traits of botanic parks. The limited body of work on park personality risks overlooking the symbolic meanings that visitors attach to their experiences in forest parks.

### 2.3 Self-congruity

Self-congruity originated from such phenomenon whereby people may impose their identity on the objects, places and others around them (Belk, 1988). In the context of place consumption, self-congruity with places connotes a perceptual matching or mismatching between a visitor’s self-concept and the stereotypic image of the place’s users (Sirgy and Su, 2000). This concept exhibits high explanatory power in accounting for visitors’ preference, choice and behavior (Sirgy, 1982).

In the field of brand marketing, it has been suggested that personified brands can help consumers legitimize their relationship with the brand (Aaker et al., 2004). In other words, brand personality can arouse consumers’ affect and feelings toward the brand. Self-congruity is considered as an effective tool for establishing and strengthening the connection between consumers and their affective bond with a brand—a relationship that further contributes to brand attachment (Swaminathan et al., 2009).

Self-congruity posits that people prefer places that exhibit characteristics consistent with their own personality and style (Sirgy et al., 1997). The basic logic of self-congruence hypothesis is rooted in the assumption that people attempt to maintain and enhance the cognitive consistency across their beliefs and behaviors (Sung and Choi, 2012). This reasoning also applies to park personality, specifically, visitors prefer those places whose image aligns with their own self-image. This implies that place-self congruity mediates the influence of destination personality on visitors’ emotional response and behavioral response.

### 2.4 Place attachment

Place attachment branches out from interdependence and attraction theories (Loureiro et al., 2012), refers to an emotional bond between individuals and specific places (Trentelman, 2009). Such places include persons’ hometowns, cities, tourism destinations, surrounding neighborhoods, and other locations (Hidalgo and Hernandez, 2001). Place attachment is an important aspect of person–place relationship, effectively embodying the strength of individuals’ connection to specific places (Ramkissoon and Mavondo, 2017). It has evolved into a complex concept that encompasses the intricate interplay among beliefs, affect, and behaviors (Kyle et al., 2005).

Numerous researchers have sought to identify the inherent components of place attachment. Ramkissoon and Mavondo (2017) suggests that place attachment involves meaningful social relationships with the local community and explains this relationship with reference to four dimensions: dependence, identity, emotion, and social connection. Scannell and Gifford (2010) proposes that place attachment is relevant to three elements: person, process, and place. Some scholars have deconstructed this concept into four components: social bonding, place affect, affective and social attachment (Plunkett et al., 2019).

### 2.5 Pro-environmental behavior

PEB represent those intentional actions that aim to minimize harm to the environments or place in both public and private settings (Juvan and Dolnicar, 2014). Individuals with strong PEB intentions spontaneously make efforts to preserve the natural environment (Stern, 2000), generate benefits for the nature (Steg and Vlek, 2009), and consciously reduce their adverse impact on environment quality (Kollmuss and Agyeman, 2002).

In research on sustainable practices in outdoor recreational settings, such as nature-based parks, a central focus for scholars and practitioners is exploring the antecedents that influence individuals’ engagement in PEB. These studies have mainly concentrated on identifying the socio-psychological factors affecting visitors’ PEB, including sensory impression and connectedness to nature (Luo et al., 2024), social interaction (Lu et al., 2024), responsibility aspiration and environment mitigation knowledge (Elsamen et al., 2025), and eco-emotion (Ágoston et al., 2024).

PEB is crucial for alleviating environmental pressure in park destination and promoting sustainable use of forest parks’ natural resources. Several theoretical frameworks have been utilized to systematically explain park visitors’ PEB decisions. These primarily include the theory of planned behavior (Esfandiar et al., 2023), the norm-activation theory (Li et al., 2025; Steg and De Groot, 2010) and value-belief-norm theory (Nguyen and Nguyen, 2021). There are three aspects of antecedents influencing park visitors’ engagement with PEB: individual, situational, and social factors. Individual factors include environmental identity (Dresner et al., 2015) and perceived connectedness to nature (Li et al., 2024). Situational factors involve park environmental facilities and perceived environment quality (Pourhossein et al., 2023). Social factors mainly refer to destination social responsibility (Pourhossein et al., 2023), community social capital (Wang, Wang, Wang, Zhang, and Liao) and social pressure from important others.

## 3 Hypothesis development

### 3.1 Destination personality and self-congruity

Research has explored how destination personality perceptions generated from on-site experience were related to visitors’ self-congruity. Matzler et al. (2016) investigated the effectiveness of brand-personification strategies and demonstrated that brand personality perceptions of tourism destinations have a positive impact on self-congruity. Chi et al. (2018) identified that destination personality perceptions specifically elicit self-congruity, which further contributes to visitors’ destination satisfaction and destination loyalty. Drawing on the theory of self-congruity, Su and Reynolds (2017) analyzed eight U.S. hotel brands. Their results indicate that the brand personality dimensions are a prominent determinant of self-image congruity and perceived functional congruity. On this basis, the following hypotheses are proposed:

*H1:* The genuineness dimensions of park personality will positively influence self-congruity.

*H2:* The competence dimensions of park personality will positively influence self-congruity.

*H3:* The sophistication dimensions of park personality will positively influence self-congruity.

*H4:* The excitement dimensions of park personality will positively influence self-congruity.

*H5:* The tranquility dimensions of park personality will positively influence self-congruity.

### 3.2 Destination personality and place attachment

The perspectives of destination personality emphasize the emotional bonds and memory association between tourists and a destination brand (Cai, 2002). Several studies suggest that brand personality reflects the emotional side of a brand (Donahay and Rosenberger, 2007). Accordingly, destination personality within tourism contexts captures tourists’ emotions and reflects the emotional appeal of a destination brand (Kim and Lehto, 2013; Papadimitriou et al., 2015).

Scholars embraced such view that destination personality is intrinsically tied to the affective component of destination image (e.g., Ekinci and Hosany, 2006; Kim and Stepchenkova, 2017; Xie and Lee, 2013). Previous research reveals that, when consuming tourism places, the affective dimensions of place image are important in fostering place attachment (Tasci et al., 2020). Huang et al. (2017), in a study conducted at a major tourist destination in China, demonstrated that perceptions of destination personality exert a positive impact on destination brand attachment. Accordingly, it can be postulated that favorable evaluations of park personality may substantially contribute to the formation of place attachment. The following hypotheses are proposed:

*H6:* The genuineness dimensions of park personality will positively influence place attachment.

*H7:* The competence dimensions of park personality will positively influence place attachment.

*H8:* The sophistication dimensions of park personality will positively influence place attachment.

*H9:* The excitement dimensions of park personality will positively influence place attachment.

*H10:* The tranquility dimensions of park personality will positively influence place attachment.

### 3.3 Self-congruity and place attachment

According to self-congruity theory, when a visitor’s self-concept aligns with a place, the visitor is more likely to develop preferences, positive emotions, and a sense of belonging toward the brand. As such, destination brand self-congruence can be conceptualized as a key driver of place attachment (Twigger-Ross and Uzzell, 1996). In leisure consumption context, the symbolic self can be consolidated and intensified, when visitors perceive destination personality as consistent with their self-concept. Consequently, self-congruity generates e emotion-engaged encounters and favorable attitudes (Sirgy and Su, 2000), which fall within the domain of place attachment.

Based on the theories of self-congruence and place attachment, Strandberg (2023), exploring the role of self-congruity as an affective trigger for resident retention, discovers a statistically significant impact on city residents’ person-place bonds and likelihood of staying. In examining the mechanism underling the relationship between self-congruity and place attachment, Pradhan et al. (2023) identified that congruity between tourists’ self-concept and destination specifically fosters destination attachment by strengthening their affective attitudes.

Park personality is closely associated with the assumption that a visitor will develop a psychological connection with a park when their own personality traits match the personality attributes of the park (Quintal et al., 2019). The formation of an emotional bond between visitors and a park is driven by the desire for social interaction and self-compatibility in navigating their environment. In this process, park visitors act as active co-creators of this relationship (Pereira et al., 2015). Previous studies have examined how the visitor self-concept aligns with the symbolic portrayals of a park, thereby shaping their attitude and behavior (Quintal et al., 2020; Quintal et al., 2021), including personal concern about the issues of environmental conservation. On this basis, it is hypothesized that:

*H11:* Self-congruity will positively influence place attachment.

### 3.4 Self-congruity and PEB

According to the hypothesis of evolutionary psychology (Ulrich, 1983), self-congruity can be comprehended as a positive psychological outcome stemming from human evolution. Not only does it help alleviate relieving mental fatigue, but it also promotes the engagement with environmentally friendly activities (Capaldi et al., 2014). In addition, the positive impact of self-congruity on PEB is grounded in the notion that individuals with a heightened sense of self-congruity allocate greater cognitive resources to directing their attention to environmental concerns, which subsequently enhances their environmental awareness and engagement (Zhang et al., 2023).

The relationship between self-congruity and environmental engagement can be explained by value-belief-norm (VBN) theory. The VBN framework suggests that individuals’ behavior is linked with personal norms, which are intrinsically motivated by values and beliefs (Pan and Zhou, 2024). According to VBN logics, the strong alignment between visitors’ self-schema and self-relevant information from the external environment generates personal norms and moral obligation (belief), which in turn lead to stronger PEB intentions. Therefore, it is hypothesized that:

*H12:* Self-congruity will positively influence PEB.

### 3.5 Place attachment and PEB

Existing research has explored how the emotional bonds with places derived from individual experience ignite PEB among individuals that support ecological sustainability. Cheng et al. (2013) found that island tourists who develop positive place emotions toward a destination are more inclined to exhibit PEB. Ramkissoon et al. (2013) further observed that the second-order construct of place attachment exerted a positive influence on PEB intentions within an Australian national park. Tonge et al. (2015), who conducted an on-site visitor survey at Ningaloo Marine Park, Australia, reported that place identity and place dependence exhibited strong positive effects on visitors’ PEB. Ram et al. (2016) identified an impact of place attachment on the perceived authenticity of tourism attractions, thereby facilitating visitors’ PEB intention. Qu et al. (2019) observed the positive influence of both affective place attachment and place dependence on the environmental engagement of mass tourists, as well as a negative effect of place identity, in a study of tourists visiting Sanya, China. The study by Tian et al. (2024) on rock-climbing tourism shows that place attachment indirectly affected individuals’ PEB intentions via the mediating role of biospheric values. A recent study by Liu et al. (2024) focused on the ecological outcomes of mountain hiking participation. Their findings indicated that place attachment is positively associated with participants’ environmentally responsible behavior. Nevertheless, empirical evidence regarding the link between place attachment and PEB remains scarce in forest park settings, especially with respect to its role in shaping visitors’ PEB. Based on the above literature, the following hypothesis is proposed:

*H13:* Place attachment will positively influence PEB.

**FIGURE 1:**
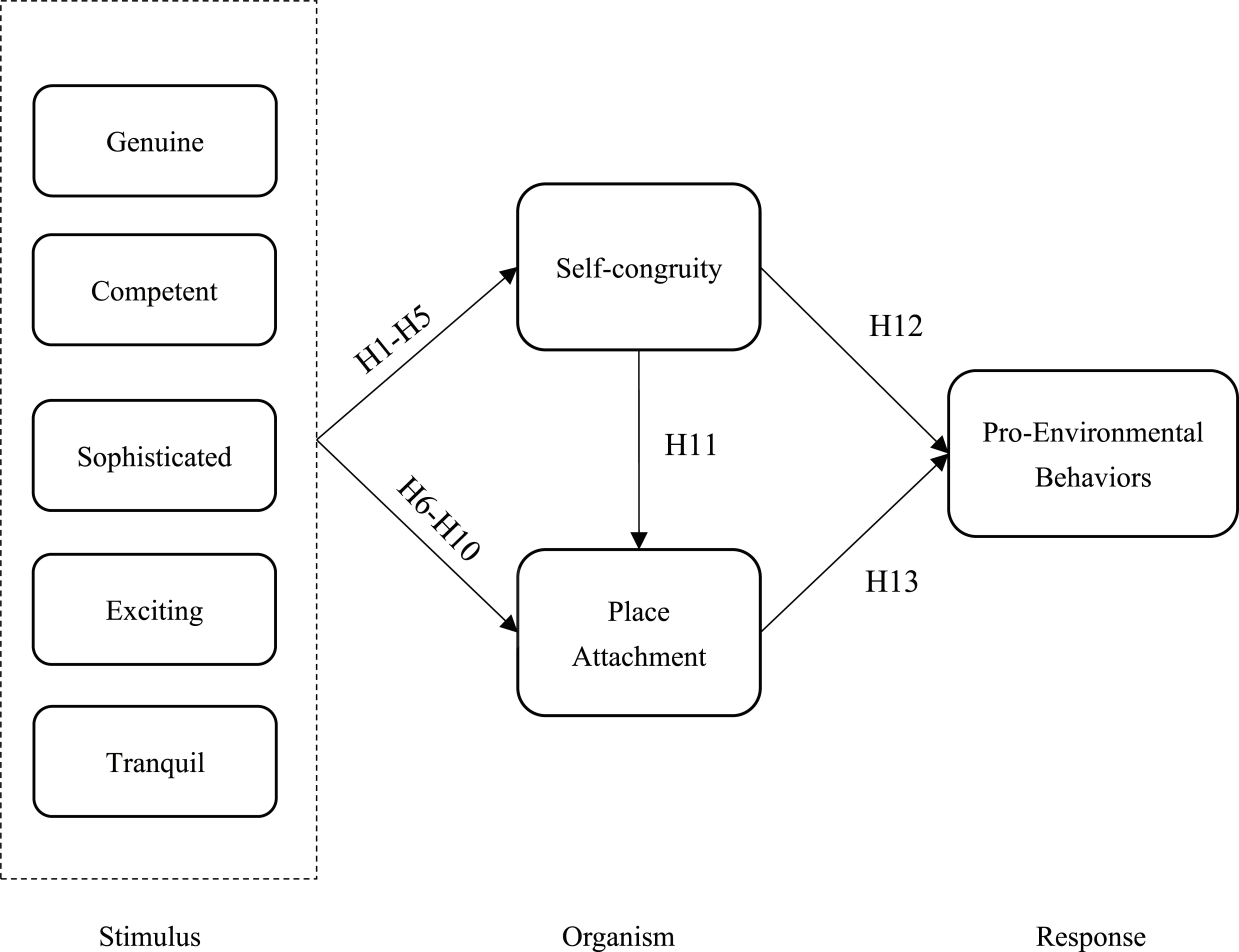
Hypothesized research model. The proposed theoretical model shows the direct and indirect effects of the constructs.

The hypotheses formulated in the preceding sections are illustrated in Figure 1, which depicts the conceptual research model.

## 4 Research methods

### 4.1 Research case site

The research context is Banshan National Forest Park (BNFP). BNFP is in Banshan street in the northeast of Gongshu District, Hangzhou City, Zhejiang Province. It is located at the southern foot of Banshan Mountain and forms a large park with Longshan Mountain and Tiger Mountain. BNFP is one of the earliest national-level forest parks within Hangzhou’s main urban area. BNFP spans 1002.88 hectares, extends over 10 kilometers, with a forest coverage rate of 90.1%. BNFP serves as a critical ecological barrier with an average negative oxygen ion concentration of 4,000 ions/cmł, which makes it recognized as an urban “green lung.” The park is one of Hangzhou’s three core ecological zones in Hangzhou, with preservation of 671 plant species, 74 bird species and 14 nationally protected animals.

**FIGURE 2:**
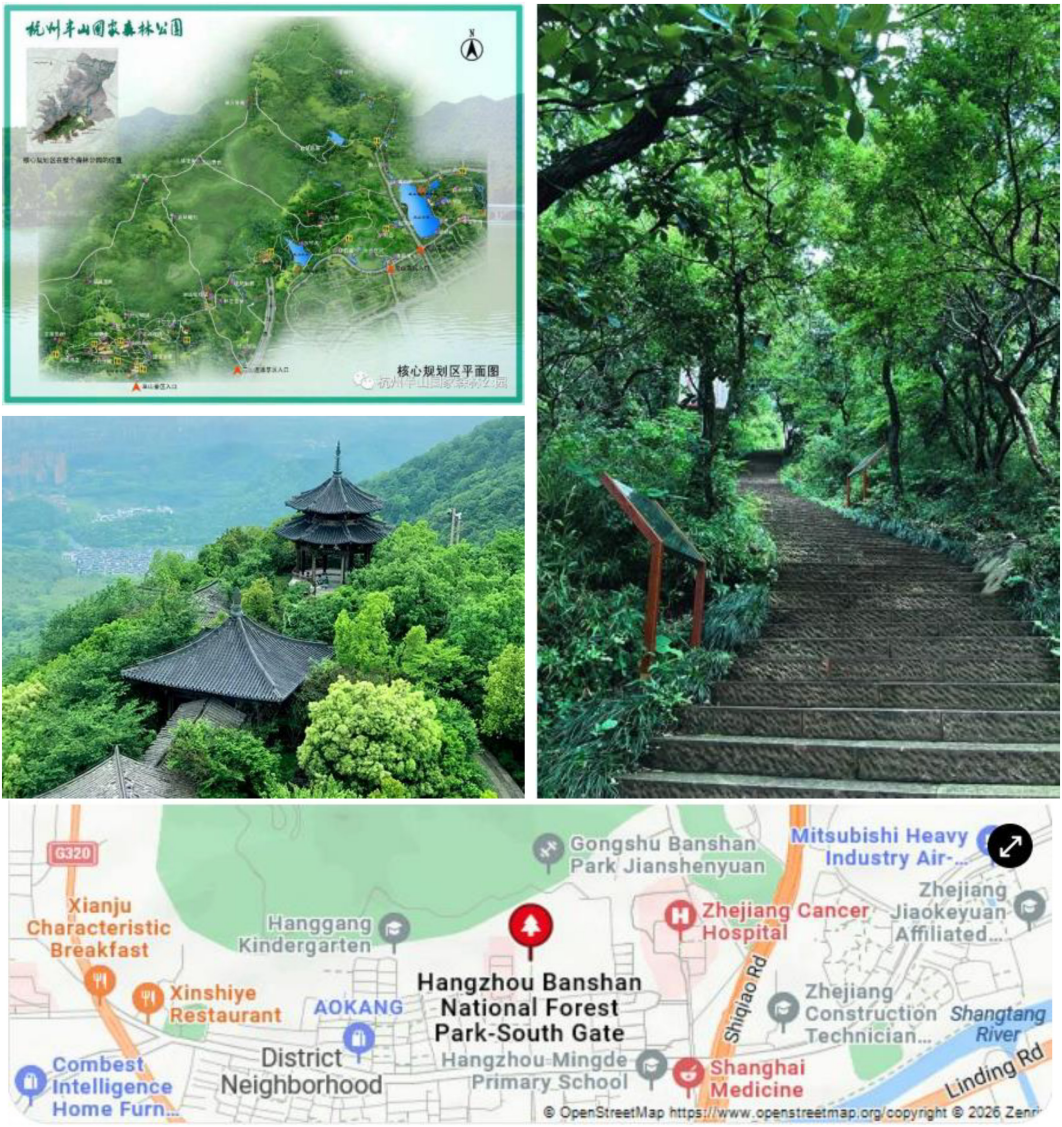
The location of BNFP and some typical images. All figures are sourced from the official WeChat account of BNFP and are used under fair use for academic purposes.

The landscape of BNFP integrates unique mountains, water resources and other natural elements of Hangzhou, as well as profound cultural heritage dating from Qin Dynasty to the present. The park features various trails that allow visitors to explore its diverse landscapes. Additionally, there are charming sculptures and gardens scattered throughout the park for visitors to enjoy. It’s a must-visit destination in Hangzhou, offering clean and tidy surroundings where visitors can appreciate the beauty of nature, including mountain views, sculpture gardens, and serene lakeside docks. Figure 2 shows the geographic location (see the image at the bottom), and guide map of BNFP (see the image on the top left side). It also presents typical image of winding trails (see the image on the right side), and ancient architectures in BNFP (see the image on the middle-left side).

BNFP is an iconic nature-based destinations in the city and attract millions of visitors annually. Selecting BNFP as the research case site provides a typical research situation for the study, which could guarantee that visitors have a prominent perception of the core concept of this study, thereby ensuring the effectiveness of variable measurement. Ant important issue for BNFP is to preserve the ecological environment and reverse environmental degradation, thereby it provided a suitable context for achieving this goal.

### 4.2 Survey instrument

Measurement items were derived from existing scales that well-established by previous studies, and reformulated slight to capture the uniqueness of BNFP (see Supplementary Appendix 1). The scales used to measure the constructs are described below.

#### 4.2.1 Destination personality

Given the significant subjectivity and circumstance dependence of destination personality assessment, prior to carrying out the survey to measure the constructs in the proposed conceptual model, it is necessary to develop a destination personality scale to systematically measure destination personality of BNFP.

First, a comprehensive review of the destination personality literature was conducted. A number of personality traits were identified from previous studies that suit the specific context of the destination under study—BNFP. Consequently, 24 destination personality items were identified from the literature.

Second, two focus groups (12 participants per group) including visitors who had visited BNFP recently were then conducted. Participants were asked to reflect on their visiting experience at BNFP and recall the emotions and feelings they had during their visitation. The questions included: (1) What characteristics do you associate with BNFP? (2) How would you describe your overall perception of BNFP using descriptive adjectives? (3) Can you provide personality metaphors to describe your impression of BNFP? And (4) If BNFP was a person, what human traits would you attribute to them? These questions encouraged participants to share their personal stories and elicit personality traits associated with rural boutique homestay. Participants share their personal stories freely and elicit personality traits associated with forest park.

A three-stage inductive content analysis was conducted on the qualitative data. Guided by trait theory, metaphor theory, three trained research assistants executed a sequential coding procedure. In the first stage, all the qualitative information were meticulously read to elicit initial codes which are relevant to destination personality traits of forest park. In the second stage, these codes were re-examined. In this process, those codes viewed as repeated and redundant were directly eliminated., while those synonymous personality characteristics were merged. This process resulted in 68 broader categories, which are accompanied by corresponding descriptions using personality metaphors. In the third stage, codes of high relevance were consolidated and promoted into elevated abstraction tiers, leading to the organization of 40 personality items. Total 64 destination personality items generated from literature review and two focus groups were further refined, causing a deletion of 30 items.

A questionnaire was developed with the remaining 34 personality trait items as key components. The face and content validity of the research tool were assessed through a focus group discussion with five participants: two managers of forest destination and three academic researchers specializing in destination personality. The instrument was pre-tested on 80 visitors to BNFP. Based on the pre-test results, two-round formal surveys were distributed using convenience sampling. An exploratory factor analysis (EFA) and a confirmatory factor analysis (CFA) were conducted to uncover the fundamental factor structure of BNFP’s personality traits and determine the reliability and validity of the measurement model identified in the EFA, respectively. Finally, a list of eighteen personality traits was identified reflecting five dimensions of BNFP’s destination personality, namely *genuine, competent, sophisticated, exciting* and *tranquil*. Those eighteen personality traits were included in the final survey.

#### 4.2.2 Self-congruity

The scale items of self-congruity were adapted from the existing literature (Matzler et al., 2016; Morhart et al., 2015; Usakli and Baloglu, 2011). This measurement solution has been applied in various contexts, such as lodging choice (Boley et al., 2022), Chinese tourists (Chen et al., 2020) and urban tourist destination (Usakli et al., 2022).

#### 4.2.3 Place attachment

Place attachment was measured through adapting the Place Attachment Inventory (PAI) developed by Williams and Vaske (2003). Four items were included in this scale. This measurement strategy has been proven to be valid in previous studies (Vada et al., 2019).

#### 4.2.4 PEB

This scale was adopted from Chiu et al. (2014) and Luo et al. (2024). Five items were included to assess tourists’ PEB intentions.

Following back-translation procedure, all measurement items were translated into Chinese to ensure translational equivalence (Brislin, 1980). To ensure the face validity and content validity of measurement items, six scholars proficient in authenticity theory, cognitive psychology and heritage management were invited to assess the clarity and relevance of the statements of each item. The resulting questionnaire also collected the socio-demographic profile of participants. A pilot test was conducted with 80 visitors of BNFP. Their feedback was addressed to further enhance the comprehension of the research instrument. The final form of the scale utilized in this study can be located in Supplementary Appendix 1. All constructs were assessed utilizing a 7-point scale (1 = completely disagree, 7 = completely agree).

### 4.3 Data collection

The formal survey was conducted during the Labor Day holiday from May 1 to 7, 2025, which was the peak visitation period. The technique of convenience sampling approach was adopted. Self-administered questionnaires were distributed to a convenience sample of visitors who had visited BNFP. Participants were intercepted at the rest areas close to the exits. An information sheet describing the core purpose of research project was presented to them. After obtaining the respondents’ permission, the questionnaires were distributed. Research assistants meticulously explain the definition of the characteristics involved in the questionnaire to ensure that participants comprehend each statement exactly. A total of 850 surveys were distributed, 785 surveys were completed and considered to be valid, for a response rate of 92.353%.

### 4.4 Analytical strategy

In the present study, PLS-SEM was applied to examine the latent constructs and test the hypotheses by using SmartPLS 3.0. PLS-SEM was chosen over CBSEM for two reasons. First, Mardia’s standardized coefficient for the measurement model (157.154) exceeded the criterion of 5 (Bentler, 2010), indicating the data violates the multivariate normality assumption. Compared to CB-SEM, PLS-SEM does not require normally distributed data, as bootstrapping technique was used to estimate standard error for its parameter estimates (Reinartz et al., 2009). Secondly, in our conceptual framework, a range of relationships should be assessed. PLS-SEM is advantageous for analyzing models containing several variables and multiple paths (Hair et al., 2011). Thirdly, sample size in this study was not very large (*N* = 785). PLS-SEM could generate higher statistical power even with lower sample sizes (Hair et al., 2019). For robust PLS-SEM estimations, the minimum sample size should be equal to 10 times the largest number of structural paths directed at a particular latent construct (Hair et al., 2014). The current sample size of 785 is adequate for PLS-SEM. Following Hair et al. (2022), a two-step analysis was conducted. Firstly, the reliability and validity of the outer model were evaluated. Then, the inner model and hypothesized path relationships were empirically assessed.

## 5 Results

A three-stage process was conducted to sequentially test the outer model, inner model and mediation effects. In the first stage, this study used the PLS regression outer model algorithm to estimate the parameters of the measurement (i.e., outer) model and latent variable (LV) scores (Kock, 2015). In the second stage, inner model criteria (e.g., measurement structural and path coefficients) are estimated based on LV scores. However, mere examination of the bivariate links between constructs may veil their true relationships due to the omission of intervening variables (Cronin et al., 2000). Therefore, in the final stage, this study explored the mediating roles of place attachment and self-congruity following the suggestion of Zhao et al. (2010). The non-parametric bootstrapping technique was used to test the significance with 785 cases and 5000 subsamples (Wells et al., 2016).

### 5.1 Descriptive data analysis

As shown in Table 1, more than one-third of the respondents were middle-aged generation aged between 36 and 45 (34.0%) and well educated with a college degree (37.6%). As for income, about half of the respondents earning RMB 5,001 to 10,000 (45.3%). Except for enterprise manager (21.4%), respondents were evenly distributed across other types of occupation. Generally, the respondents were extracted from different social groups, and thus had desirable representativeness. Descriptive statistics for survey items are presented in Supplementary Appendix 1. Normality tests also show that some items have skewness and kurtosis above the required cut-off points of -1 and + 1, revealing that the data exhibited a non-normal distribution (Hair et al., 2022).

**TABLE 1:** Demographic profile of the respondents (*N* = 785).

Characteristics	N	%	Characteristics	N	%
**Gender**			**Age**		
Male	391	49.8	18–25	127	16.2
Female	394	50.2	26–35	180	22.9
**Education**			36–45	267	34.0
Primary school	45	5.7	46–55	123	15.7
Middle school	141	18.0	56–65	44	5.6
High school	166	21.1	> 65	44	5.6
College	295	37.6	**Persona monthly income (RMB)**		
Post-graduate	138	17.6	3,000 and below	236	30.1
**Occupation**			3,001–5,000	49	6.2
Student	233	29.7	5,001–7,000	190	24.2
Enterprise staff	127	16.2	7,001–10,000	166	21.1
Enterprise manager	168	21.4	10,001–15,000	112	14.3
Private business owner	98	12.5	15,001–20,000	4	0.5
Government staff/civil servant	85	10.8	20,001 and above	28	3.6
Retired	31	3.9			
Freelance	37	4.7			
Others	6	0.8			

PMI, Persona monthly income.

### 5.2 Outer model results

Simultaneously measuring the destination personality, place attachment, self-congruity and PEB of the respondents may lead to a common response deviation when answering the questionnaire. For this purpose, an alternative method proposed by Liang et al. (2007) was referred as a test criterion for this common method bias. The result demonstrates that the average substantively explained variance of the indicators is 0.774(Ra^2^), while the average method-based variance is 0.006 (Rb^2^) (see Supplementary Appendix 2). The ratio of Ra^2^ to Rb^2^ is 136.869. Moreover, most of the method factor loadings are negative and insignificant. Hence, common method bias is not a serious concern.

Confirmatory factor analysis (CFA) was conducted to assess the reliability, and the convergent and discriminate validity of the outer model, which follows the procedure suggested by Fornell and Larcker (1981). As shown in Table 2, the results first upheld the convergent validity of all constructs, as each measurement item for eight reflective constructs exceeded the recommended value of 0.708 (Hair et al., 2021). Internal reliability of all the constructs was established, with Cronbach’s alpha, rho_A, and CR values exceeding the minimum threshold of 0.7 (Nunnally and Bernstein, 1994). The average variances extracted (AVE) values exceeded the required 0.5 threshold, suggesting acceptable convergent validity was achieved.

**TABLE 2:** Summarized results of measurement properties of outer model evaluation.

Constructs	Items	Loading	Cronbach’s α	rho_A	CR	AVE
Genuine	GEN1	0.867	0.857	0.858	0.913	0.778
	GEN2	0.895				
	GEN3	0.884				
Competent	COM1	0.901	0.894	0.895	0.934	0.825
	COM2	0.921				
	COM3	0.903				
Sophisticated	SOP1	0.844	0.897	0.898	0.929	0.765
	SOP2	0.893				
	SOP3	0.875				
	SOP4	0.885				
Exciting	EXC1	0.874	0.911	0.912	0.937	0.789
	EXC2	0.904				
	EXC3	0.900				
	EXC4	0.875				
Tranquil	TRA1	0.826	0.895	0.896	0.927	0.761
	TRA2	0.904				
	TRA3	0.881				
	TRA4	0.875				
Self-congruity	SC1	0.899	0.884	0.885	0.928	0.811
	SC2	0.910				
	SC3	0.894				
Place attachment	PA1	0.855	0.874	0.875	0.914	0.726
	PA2	0.884				
	PA3	0.829				
	PA4	0.841				
Pro-environmental behavior	PEB1	0.880	0.923	0.923	0.942	0.764
	PEB2	0.857				
	PEB3	0.890				
	PEB4	0.879				
	PEB5	0.862				

The results also uphold discriminant validity, as the square roots of AVE scores for each construct were higher than the corresponding cross-variable correlations (Do Valle and Assaker, 2016; Table 3). Discriminant validity was also assessed by the heterotrait-monotrait ratio of correlations (HTMT) values. The calculated value of HTMT ratio calculated in this study were below the threshold of 0.9, also implying acceptable discriminant validity (Hair et al., 2021).

**TABLE 3:** Assessment of the discriminant validity using Fornell-Larcker criterion and HTMT technique.

	GEN	COM	SOP	EXC	TRA	SC	PA	PEB
Fornell and Larcker criterion								
Genuine	0.882	0.908	0.875	0.888	0.872	0.901	0.852	0.874
Competent	0.565							
Sophisticated	0.596	0.787						
Exciting	0.639	0.730	0.774					
Tranquil	0.575	0.640	0.754	0.726				
Self-congruity	0.688	0.578	0.618	0.647	0.620			
Place attachment	0.557	0.673	0.721	0.762	0.759	0.620		
PEB	0.591	0.649	0.729	0.784	0.790	0.636	0.802	
Hetero-trait-mono-trait ratio (HTMT)								
Genuine	0.644	0.879	0.854	0.802	0.696	0.706	0.892	
Competent								
Sophisticated	0.678							
Exciting	0.722	0.808						
Tranquil	0.656	0.714	0.839					
Self-congruity	0.789	0.650	0.692	0.720				
Place attachment	0.644	0.762	0.813	0.853	0.857			
PEB	0.664	0.714	0.800	0.854	0.868	0.704		

Diagonally positioned values in bold denotes the square roots of AVEs.

### 5.3 Inner model results

**FIGURE 3:**
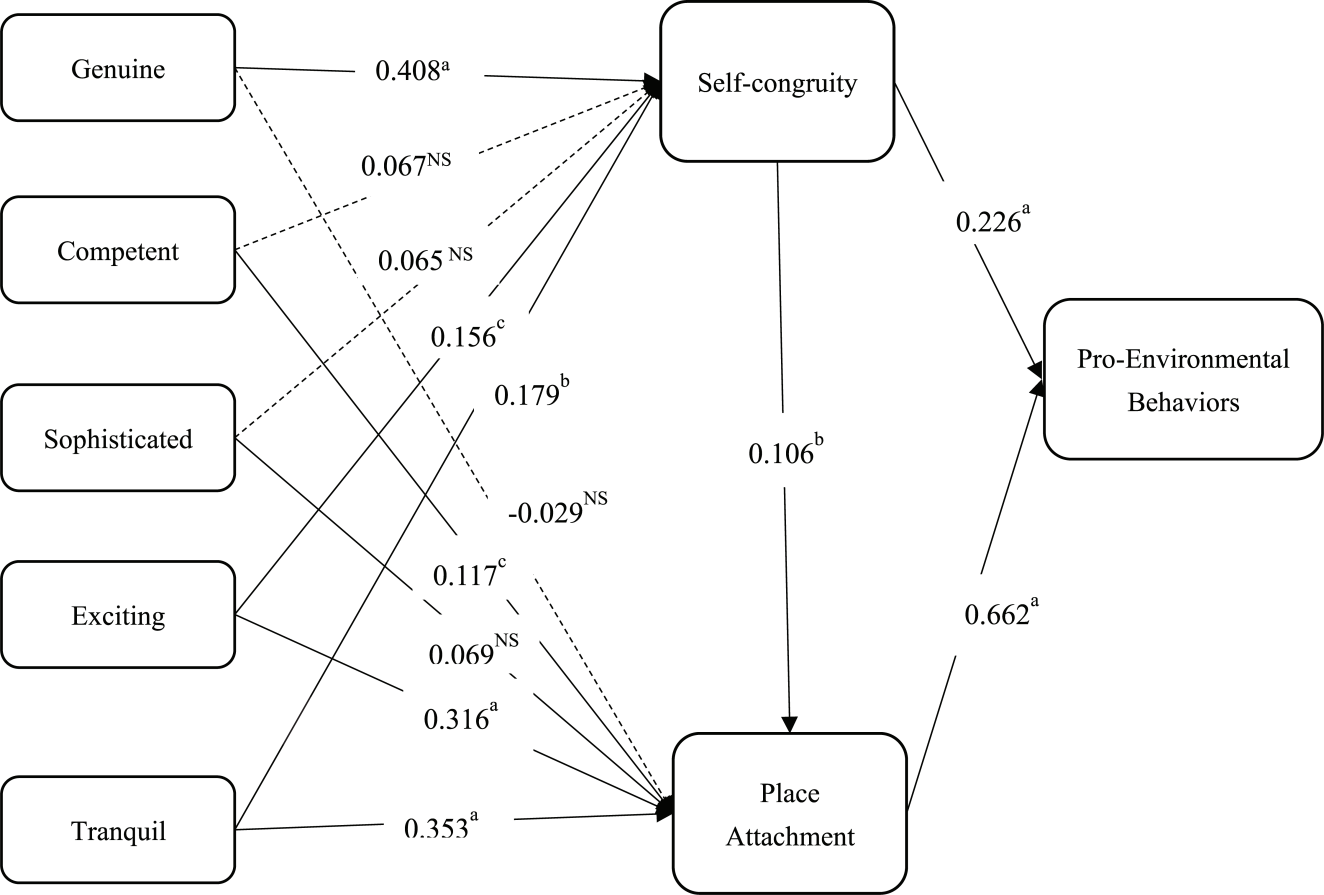
Results of the inner model evaluation. ^a^*p <* 0.001; ^b^*p <* 0.01; ^c^*p <* 0.05; ^NS^*p >* 0.05.

Figure 3 and Table 4 presents the results of the inner model assessment. The variance inflation factors (VIFs) based on each path were yielded, none of which exceeded the reference value of 5.00 (Hair et al., 2019). This finding indicates that no potential multicollinearity problem exists. The bootstrapping with 5,000 subsamples were performed to calculate the path coefficients.

**TABLE 4:** Result of structural path analysis and hypothesis tests (inner model evaluation).

Hypothesis	Predicted relationships	Path coefficient	Standard error	*T*-value	Percentile 95% CI		VIF	Test results
					Lower	Upper		
H1	GEN→SC	0.408^a^	0.043	9.497	0.32	0.487	1.817	Support
H2	COM→SC	0.067^NS^	0.051	1.330	−0.035	0.163	2.935	Reject
H3	SOP→SC	0.065^NS^	0.059	1.108	−0.048	0.182	3.991	Reject
H4	EXC→SC	0.156^c^	0.062	2.511	0.042	0.285	3.304	Support
H5	TRA→SC	0.179^b^	0.056	3.188	0.068	0.288	2.672	Support
H6	GEN→PA	−0.029^NS^	0.035	0.829	−0.092	0.043	2.205	Reject
H7	COM→PA	0.117^c^	0.057	2.077	0.004	0.226	2.946	Support
H8	SOP→PA	0.069^NS^	0.060	1.140	−0.046	0.191	4.001	Reject
H9	EXC→PA	0.316^a^	0.052	6.107	0.214	0.415	3.361	Support
H10	TRA→PA	0.353^a^	0.047	7.586	0.261	0.445	2.747	Support
H11	SC→PA	0.106^b^	0.04	2.629	0.026	0.181	2.337	Support
H12	SC→PEB	0.226^a^	0.032	7.000	0.164	0.289	1.625	Support
H13	PA→PEB	0.662^a^	0.030	22.370	0.602	0.715	1.625	Support

^a^*p <* 0.001;

^b^*p <* 0.01;

^c^*p <* 0.05; ^NS^*p >* 0.05; CI, confidence interval; 5,000 bootstrap samples.

The results support the hypothesized positive relationships between genuine (β_Ha1_ = 0.408, *p* < 0.001), exciting (β_Ha4_ = 0.156, *p* < 0.05), tranquil (β_Ha5_ = 0.179, *p* < 0.01), and self-congruity. Ha1, Ha4, and Ha5 are supported. However, the paths from competent (β_Ha2_ = 0.067, *p >* 0.05) and sophisticated (β_Ha3_ = 0.065, *p >* 0.05) to self-congruity are not significant. Ha2 and Ha3 are rejected.

Also, Ha7, Ha9, and Ha10 are supported, i.e., place attachment is significantly influenced by competent (β_Ha7_ = 0.117, *p* < 0.05), exciting (β_Ha9_ = 0.316, *p* < 0.001), and tranquil (β_Ha10_ = 0.353, *p* < 0.001). However, the paths from genuine (β_Ha6_ = -0.029, *p* > 0.05) and sophisticated (β_Ha8_ = 0.069, *p* > 0.05) to place attachment are not significant. Ha6 and Ha8 are rejected.

Self-congruity was found to positively influence place attachment (β_Ha11_ = 0.106, *p* < 0.01) and PEB (β_Ha12_ = 0.226, *p* < 0.001), lending support to Ha11 and Ha12. Place attachment was positively associated with PEB (β_Ha13_ = 0.662, *p* < 0.001); hence, Ha13 was supported.

### 5.4 Mediating effect test

We also explored the potential mediating effect of self-congruity and place attachment. Findings appear in Table 5. Bootstrapping procedure was applied to set repeated sampling 5,000 times, and output the confidence interval (CI). There is a significant indirect effect when the bootstrap confidence interval does not include zero.

**TABLE 5:** Indirect paths from park personality dimensions to PEB (mediating roles of SC and PA).

Predicted relationships	Point estimate	Standard error	*p*	Percentile 95% CI		BC 95% CI	
				Lower	Upper	Lower	Upper
GEN→SC→PEB	0.092	0.016	< 0.001	0.062	0.125	0.063	0.126
COM→SC→PEB	0.015	0.012	0.189	−0.008	0.038	−0.007	0.039
SOP→SC→PEB	0.015	0.014	0.285	−0.011	0.044	−0.011	0.044
EXC→SC→PEB	0.035	0.015	0.019	0.009	0.067	0.009	0.068
TRA→SC→PEB	0.041	0.015	0.005	0.014	0.071	0.015	0.073
GEN→PA→PEB	−0.019	0.023	0.407	−0.061	0.028	−0.064	0.026
COM→PA→PEB	0.078	0.037	0.036	0.003	0.149	0.003	0.150
SOP→PA→PEB	0.046	0.040	0.253	−0.031	0.125	−0.032	0.123
EXC→PA→PEB	0.209	0.038	< 0.001	0.136	0.284	0.137	0.284
TRA→PA→PEB	0.234	0.034	< 0.001	0.169	0.301	0.171	0.304

Un-standardized estimates are reported; CI, confidence interval; BC, bias-corrected bootstrap; 5,000 bootstrap samples.

Two groups of models were constructed. The first group of models contained five destination personality factors and PEB with self-congruity as the mediator; the second group of models examined relationships between PEB and park personality factors using place attachment as the mediator.

For the first group of models, the indirect effects of genuine, exciting and tranquil on PEB through self-congruity were supported by significant mediations, with an estimate of 0.092 (*p* < 0.001), 0.035 (*p* < 0.05), and 0.041 (*p* < 0.01), respectively. The indirect effects of competent and sophisticated on PEB through self-congruity were not significant with an estimate of 0.015 (*p* = 0.189) and 0.015 (*p* = 0.285).

For another group of models, the indirect effects of competent, exciting, and tranquil on PEB through place attachment were supported by significant mediations, with an estimate of 0.078 (*p* < 0.05), 0.209 (*p* < 0.001), and 0.234 (*p* < 0.001), respectively. The indirect effects of genuine and sophisticated on PEB through place attachment were not significant with an estimate of -0.019 (*p* = 0.407) and 0.046 (*p* = 0.253).

## 6 Discussion and conclusion

The current study integrates the stimulus-organism-response (SOR) model and destination personality theory to predict visitors’ PEB intentions in the context of forest parks. It has been widely accepted that destination personality is helpful in crafting unique destination identity and image, owing to its distinctiveness and non-substitutability (Novais et al., 2018). This study investigates the roles of park personality as an essential environmental stimulus, as well as the mediating effects of place attachment and self-congruity, in driving visitors to make and implement PEB decision. The results of this study can be synthesized as follows.

First, this study reveals that different dimensions of park personality exhibit distinctive effects on visitors’ self-congruity. Specifically, genuine, exciting, and tranquil traits were directly and positively related to self-congruity (H1, H4, and H5 were supported). Accordingly, compared with the other two dimensions, these three factors serve a more critical function in fostering psychological consistency between visitors’ self-concept and the image of park destinations. This finding aligns with prior work by Usakli and Baloglu (2011), Matzler et al. (2016), and Chi et al. (2018). Notably, in contrast to genuine, exciting, and tranquil, competent and sophisticated dimensions exhibited no direct linkage with self-congruity (H2 and H3 were not supported). A majority of forest visitors are driven by desires for unspoiled natural scenery (genuine), hedonic experiences (exciting), and inner peace (tranquil) (Oppliger et al., 2019). Consequently, park visitors are more inclined to associate their self-identity with genuine, exciting, and tranquil, rather than competent or sophisticated. Regarding competent, the present findings diverge from previous research (Souiden et al., 2017) where urban destinations’ competence positively influences tourist behavior. One potential explanation underpinning this discrepancy is that competence represents a more cognitively and functionally oriented destination attribute; visitors to BNFP are less attentive to this dimension and less likely to link it to their self-concept (Huaman-Ramirez et al., 2023). In this regard, this study advances existing literature by conceptualizing destination personality as a five-dimensional construct and differentiating the heterogeneous impacts of its sub-dimensions on self-congruity.

Secondly, the results suggest that the distinct dimensions of park personality play differential roles in shaping visitors’ place attachment. The relationships between destination personality dimensions and place attachment were largely significant, with only two exceptions. Specifically, competent, exciting, and tranquil emerge as the most prominent personality traits in fostering emotional bonding between visitors and places (H7, H9, and H10 were supported). In this study, place attachment was used to capture park visitors’ internal psychological state as the organism response in SOR theory (Qiu et al., 2023). This finding is consistent with prior research, which indicate that destination personality constitutes the pivotal factor in predicting the positive affective bond that visitors establish with a tourism place (Xie and Lee, 2013). This finding also echoes the observation that personification of destination elicits visitors’ identification with the unique symbolic attributes of the place, thereby facilitating the development of a strong emotional bond with it (Blain et al., 2005).

In contrast, genuine and sophisticated exert no influence on place attachment (H6 and H8 were not supported). Accordingly, these two traits were not directly associated with visitors’ emotional bonds with forest parks. These two insignificant relationships partially contradict the conceptual model, which assumes consistent effects of personality dimensions. This contradictory finding may be explained by the fact that more than 50% of respondents in this study were aged between 26 and 45. These young adults are active in the workforce and perceive forest park as a sanctuary enabling them to temporarily escape socially imposed roles (Thomassen, 2012). They tend to regard park visits as an opportunity to engage in stimulating activities (exciting) or psychological detachment (tranquil). The parks’ ability to provide satisfactory services and high-quality infrastructures is also important to them (competence). Consequently, young visitors are likely to develop strong emotional bonds with forest parks due to the personality-related perceptions of excitement, sophistication, and competence. Genuine and sophisticated represent the unspoiled natural environments and aesthetic value of park destination, respectively. These attributes do not align closely with young visitors’ psychological need, thus contributing little to the formation of emotional bonding between visitors and park destinations.

Thirdly, the mediation analysis, designed to unveil the true relationships among constructs, revealed that self-congruity significantly mediates the relationship between the three personality traits (genuine, exciting, and tranquil) and visitors’ PEB intentions. These three traits were further identified as the most salient predictors of visitors’ self-congruity. This finding implies that self-congruity serves as a critical factor in mediating the impact of park personality on PEB. The above findings can be interpreted through the lens of self-expression theory. Destination personality encapsulates the self-expressive benefits that visitors derive from the destinations (Shi et al., 2026). Relative to the personality dimensions of competent and sophisticated, park visitors are more likely to adopt the aforementioned three traits as “consumption symbols” to express themselves (Sirgy, 1982) and engage in PEB. The mediation role of self-congruity is also supported by attention restoration theory. Forest parks are venues for physical and psychological refreshment (Zhou et al., 2025). Stronger perceptions of park personality indicate that the destination exerts a restorative effect that cultivates visitors’ sense of self-consistency and self-enhancement. These processes further strengthen visitors’ sense of responsibility regarding adverse environmental impacts.

Finally, the study further highlights the role of place attachment as a mediator between three dimensions of park personality (competent, exciting, and tranquil) and visitors’ PEB intention. These three traits were also identified as the most important factors shaping visitors’ place attachment. This finding suggests that place attachment represents another critical mediating mechanism park personality to PEB. These results also confirmed the importance of place attachment in park visitation, as it bridges the relationships between park personality and PEB. The mediating effect of place attachment uncovers an alternative mechanism of need fulfillment through which park personality promotes visitors’ PEB: perceived destination personality enables visitors to identify with a destination’s unique symbolic and emotional attributes, thereby fostering strong emotional bond with the destination and a greater intention to engage in PEB. The psychological mechanisms underpinning the mediation role of place attachment can be further interpreted from the perspective of visitors’ affective bonding. Rather than merely reflecting a physical or functional image, destination personality represents an affective component of the tourism destination (Murphy et al., 2007a). It is intrinsically linked to the emotional elements of destination image, which evoke affective bonds among park visitors. These responses are transformed into place attachment and subsequently generate spontaneous actions taken to protect the natural environment.

## 7 Implications and limitations

The findings enrich our understanding of the psychological mechanism underlying forest park visitors’ PEB intention in several ways, particularly highlighting the neglected role of destination personality in the context of forest parks. The unique appeal of park destinations resides in their provision of opportunity for outdoor recreation and a variety of nature-based activities (Konu, 2015). The concept of destination personality encapsulates the intrinsic and symbolic meanings embedded in park visitors’ experiences (Shi et al., 2025). Nonetheless, empirical research on park personality remains limited. The symbolic attributes of forest parks have been insufficiently explored, let alone their potential impacts on visitors’ PEB intention. Accordingly, this study carries both theoretical and practical implications.

### 7.1 Theoretical implications

First, conducted in a representative forest park, this study explores the interactive relationships among destination personality, place attachment, and self-congruity that influence visitors’ PEB intention. Destination personality has rarely been incorporated into research on visitors’ engagement in on-site PEB; thus, an important contribution of this study is enhancing our understanding of how park visitors make on-site PEB decisions. This new perspective provides a theoretical foundation for further exploring the factors influencing visitors’ on-site PEB. This study confirms that it is crucial to emphasize the role of the destinations’ symbolic attributes in addressing visitors’ PEB intention. The finding that forest park can be characterized by five-component personality traits could be regarded as a supplementary route to identifying green consumption intentions in park context from an affective-symbolic standpoint.

Secondly, an important contribution of this research is the identification of the five dimensions of destination personality exhibited by BNFP. This study focuses specifically on forest park, a distinct category of natural destinations. While previous research on ecotourism, park destinations, and forests has been insightful, visitors’ perceptions of forest parks’ destination personality remain understudied. Among the traditional five dimensions of brand personality proposed in Aaker (1997) framework, sincerity, excitement, competence and sophistication are found to be applicable to BNFP. Tranquility is consistent with findings from other destination personality studies (Hu et al., 2020). This research thus advances beyond existing studies by developing a systematic framework for examining destination personality in the context of forest park visit. The five-dimensional scale can serve as a research tool in future studies to assess how visitors from different cultural backgrounds evaluate parks in natural areas.

Thirdly, the context-specific dimensions of forest parks’ destination personality reveal that each destination is described by dispositional traits; hence a context-specific and culturally appropriate measurement scale for assessing destination personality may need to be developed. This finding also echoes the emic-etic assumption, which posits that destination personality comprises generalizable dimensions across diverse sociocultural contexts, as well as culture-specific dimensions tied to specific contexts (e.g., Cheung et al., 1996; Sahin and Baloglu, 2011). For instance, genuineness—which connotes “destination sincerity”— justifies the “sincerity” dimension of the BPS within an Eastern cultural context. Tranquility, meanwhile, symbolizes visitors’ spiritual yearning for seclusion and simplicity. For these visitors, the forest park functions as a utopian retreat that possesses restorative potential to help visitors alleviate physical and mental fatigue. Collectively, these results indicate that cultural context exerts a profound influence on the construction of individuals’ destination personality perceptions. This finding supports the proposition that the meanings carried by certain tourism products are shaped by both culturally universal and context-specific factors (Aaker et al., 2001).

### 7.2 Practical implications

The findings of this study have practical values for promoting sustainable forest park management. First, this study extends existing research by conceptualizing forest park destination personality as a five-dimensional construct and differentiating the distinctive effects of its underlying dimensions (i.e., genuine, competent, sophisticated, exciting, and tranquil). This finding can help park marketers determine the most important park personality attributes that drive visitors’ favorable behaviors. It also provides guidance for resource allocations and service-scape design, which are vital to the sustainability of the parks’ ecological environment. Among the five dimensions of park personality, exciting and tranquil are influential factors in shaping both place attachment and self-congruity, which in turn positively influence visitors’ PEB. Thus, this attribute could serve as a key reference for forest parks’ environmental protection strategies. Park operators can implement nature-based activities that cater to visitors’ desire to enjoy the peace of mind and seek spiritual solitude in the natural environment. They can also launch winding trails, secret gardens, and discovery play areas to extend their offerings and provide opportunities for public to engage in immersive visitation experience. Additionally, they can organize a series of sedentary and vibrant activities to cultivate visitors’ perception of its personality traits of tranquil and exciting.

Second, given the prominent role of place attachment in shaping visitors’ PEB intention, it is recommended to enhance visitors’ emotional attachment by strengthening the parks’ capacity to address visitors’ deeper psychological demands. Respondents who perceive a strong park personality will develop place attachment to the park via its softer aspect of destination attractiveness, which further reinforce their PEB intention. Therefore, managers should fully consider the demand preferences of park visitors when reconfiguring open green spaces and assessing whether visitors value the destination attractiveness conveyed by the ecological environment. The intimate association between place attachment and PEB indicates that the design of park landscapes, routes, and activities should be optimized to better strengthen the visitors’ emotional resonance with the park and encourage them to form place attachments. Positive relationships exist between self-congruity and PEB. By understanding how visitors perceive parks’ destination personality, park managers can cultivate and enhance the internal dimensions of park identity and conduct targeted promotional efforts based on the core five factors of park personality to highlight the distinctive characteristic of parks’ recreational offerings. Such practices have the potential to enable visitors more readily identify with the park and establish self-congruity, which can be converted into visitors’ PEB decisions.

### 7.3 Limitations and future research

The limitations inherent in this study include five aspects. First, data were collected from one forest park located in Hangzhou, China. This restricts the generalizability of the findings obtained by this study. Future research could collect larger samples from multiple sites. Comparative studies with other types of park destinations could be conducted to enhance the generalizability of the results.

Secondly, since little previous research has investigated visitors’ perceptions of forest parks’ destination personality, most items in the current study were selected from prior studies conducted in other cultural contexts. The measurement items may not be sufficiently comprehensive to cover all dimensions of park personality of forest parks, even though in-depth interviews were conducted. More qualitative approaches could be employed in future research to develop a more comprehensive item pool and achieve a thorough understanding of the brand personality of Chinese forest parks. This would help identify the key destination personality traits perceived by actual visitors to forest parks.

Thirdly, the application of structural equation modeling (SEM) has certain limitations. Further investigations into PEB, particularly when exploring its subtle influencing mechanisms, require the integration of multiple research methods.

Fourthly, this study only focuses on the direct relationships between PEB and its antecedents; accordingly, future efforts could be made to add incremental explanatory and predictive power of the findings. For instance, future research may incorporate moderating boundaries of destination personality- PEB relationship, such as individuals’ demographic characteristics, environmental values, belief, and environmental knowledge.

Finally, this study was conducted during the summer months. Given the distinct four-season climate of Hangzhou, future research could explore whether seasonal variations would influence visitors’ perceptions toward park personality and their PEB intention.

## Data Availability

The raw data supporting the conclusions of this article will be made available by the authors, without undue reservation.
