# Supplementary material for: The effect of destination personality dimensions on park visitors’ pro-environmental behavior: an application of stimulus-organism-response model
Source: Front Psychol. 2026 Apr 2;17:1734312. doi: 10.3389/fpsyg.2026.1734312 (PMC13083017; doi:10.3389/fpsyg.2026.1734312)
Supplement: Supplementary file 1 [file Table_1.docx]

Appendix A. Survey items and descriptive statistics

| Constructs and Items | Mean ± SD | Skewness | Kurtosis |
| --- | --- | --- | --- |
| **Destination personality1-Genuine** | | | |
| GEN1.This park is original. | 5.217±1.135 | -0.663 | 1.010 |
| GEN2. This park is authentic. | 5.222±1.165 | -0.657 | 0.866 |
| GEN3. This park is real. | 5.233±1.148 | -0.581 | 0.740 |
| **Destination personality2-****Competent** | | | |
| COM1.This park is successful. | 5.098±1.177 | -0.615 | 1.088 |
| COM2. This park is well-made. | 5.200±1.099 | -0.662 | 1.247 |
| COM3. This park is satisfying. | 5.154±1.115 | -0.523 | 0.875 |
| **Destination personality-****Sophisticated** | | | |
| SOP1. This park is good-looking. | 5.245±1.077 | -0.658 | 1.401 |
| SOP2. This park is classy. | 5.279±1.110 | -0.909 | 1.937 |
| SOP3. This park is glamorous. | 5.205±1.124 | -0.594 | 1.006 |
| SOP4. This park is stylish. | 5.273±1.098 | -0.619 | 1.202 |
| **Destination personality- Exciting** | | | |
| EXC1. This park is spirited. | 5.219 ±1.125 | -0.794 | 1.287 |
| EXC2. This park is energetic. | 5.171 ±1.057 | -0.429 | 0.647 |
| EXC3. This park is playful. | 5.238 ±1.063 | -0.588 | 0.899 |
| EXC4. This park is free. | 5.254 ±1.065 | -0.443 | 0.551 |
| **Destination personality-Tranquil** | | | |
| TRA1. This park is calm. | 5.141±1.099 | -0.398 | 0.329 |
| TRA2. This park is peaceful. | 5.138±1.120 | -0.541 | 0.716 |
| TRA3. This park is relaxing. | 5.078±1.137 | -0.502 | 0.562 |
| TRA4. This park is reclusive. | 5.167±1.126 | -0.407 | 0.583 |
| **Self-Congruity** |  |  |  |
| SC1. The personality of this park is consistent with how I see myself. | 5.111±1.157 | -0.460 | 0.513 |
| SC2. The personality of this park reflects mine. | 5.130±1.150 | -0.538 | 0.415 |
| SC3. The personality of this park is close to my own personality. | 4.971±1.176 | -0.292 | -0.074 |
| **Place Attachment** |  |  |  |
| PA1. I feel that this park is a part of me. | 5.270±1.101 | -0.682 | 1.268 |
| PA2. This park is the best place for what I like to do. | 5.284±1.120 | -0.669 | 1.258 |
| PA3. This place is very special to me. | 5.015±1.261 | -0.653 | 0.714 |
| PA4. No other place can compare to this place. | 5.175±1.157 | -0.671 | 1.111 |
| **Pro-Environmental Behavior** |  |  |  |
| PEB1. I always take a more sustainable approach during visitation, such as handling the rubbish carefully, etc. | 5.092±1.123 | -0.587 | 0.898 |
| PEB2. I respect the environment of the destination. | 5.104±1.092 | -0.497 | 0.414 |
| PEB3. I will discuss the environmental protection issues of the destination with my fellow travelers. | 5.149±1.102 | -0.567 | 0.890 |
| PEB4. I will try to persuade my family and friends to adopt environmentally friendly behaviors. | 5.028±1.217 | -0.539 | 0.485 |
| PEB5. When I see someone damaging the environment in the area, I will stop it. | 5.033±1.148 | -0.415 | 0.453 |

Appendix B. Test for common method bias.

| Construct | Indicators | Substantive factor loading (Ra) | Substantive variance (Ra^2^) | Method factor loading (Rb) | Method variance (Rb^2^) |
| --- | --- | --- | --- | --- | --- |
| Genuine | GEN1 | 0.868 | 0.753 | -0.078 | 0.006 |
|  | GEN2 | 0.895 | 0.801 | -0.076 | 0.006 |
|  | GEN3 | 0.883 | 0.780 | 0.037 | 0.001 |
| Competent | COM1 | 0.899 | 0.808 | 0.209 | 0.044 |
|  | COM2 | 0.920 | 0.846 | -0.009 | 0.000 |
|  | COM3 | 0.906 | 0.821 | -0.041 | 0.002 |
| Sophisticated | SOP1 | 0.837 | 0.701 | -0.067 | 0.004 |
|  | SOP2 | 0.896 | 0.803 | 0.054 | 0.003 |
|  | SOP3 | 0.880 | 0.774 | 0.019 | 0.000 |
|  | SOP4 | 0.885 | 0.783 | -0.014 | 0.000 |
| Exciting | EXC1 | 0.869 | 0.755 | -0.063 | 0.004 |
|  | EXC2 | 0.904 | 0.817 | -0.102 | 0.010 |
|  | EXC3 | 0.903 | 0.815 | -0.046 | 0.002 |
|  | EXC4 | 0.877 | 0.769 | -0.136 | 0.018 |
| Tranquil | TRA1 | 0.827 | 0.684 | 0.100 | 0.010 |
|  | TRA2 | 0.906 | 0.821 | -0.048 | 0.002 |
|  | TRA3 | 0.874 | 0.764 | 0.085 | 0.007 |
|  | TRA4 | 0.880 | 0.774 | 0.066 | 0.004 |
| Self-Congruity | SC1 | 0.896 | 0.803 | 0.039 | 0.002 |
|  | SC2 | 0.911 | 0.830 | -0.154 | 0.024 |
|  | SC3 | 0.896 | 0.803 | 0.054 | 0.003 |
| Place Attachment | PA1 | 0.855 | 0.731 | 0.009 | 0.000 |
|  | PA2 | 0.884 | 0.781 | 0.066 | 0.004 |
|  | PA3 | 0.827 | 0.684 | 0.012 | 0.000 |
|  | PA4 | 0.842 | 0.709 | 0.057 | 0.003 |
| Pro-Environmental Behavior | PEB1 | 0.878 | 0.771 | -0.034 | 0.001 |
|  | PEB2 | 0.859 | 0.738 | -0.026 | 0.001 |
|  | PEB3 | 0.890 | 0.792 | 0.078 | 0.006 |
|  | PEB4 | 0.880 | 0.774 | -0.016 | 0.000 |
|  | PEB5 | 0.862 | 0.743 | 0.008 | 0.000 |
| Average |  |  | 0.774 |  | 0.006 |

Note: 5000 bootstrap samples
